# Motivational Interventions in Prenatal Clinics

**Published:** 2001

**Authors:** Nancy Sheehy Handmaker, Paula Wilbourne

**Affiliations:** Nancy Sheehy Handmaker, Ph.D., is a research assistant professor and principal investigator of a National Institute on Alcohol Abuse and Alcoholism-funded project, “Motivating Pregnant Drinkers” and Paula Wilbourne, M.S., is a doctoral student in the Department of Psychology, University of New Mexico, Albuquerque, New Mexico

**Keywords:** motivational interviewing, brief intervention, prevention, prenatal care, pregnancy, prenatal alcohol exposure, fetal alcohol syndrome, treatment outcome, health care delivery

## Abstract

Although the risks associated with pregnancy are well-documented, prevention efforts, for the most part, have not reached women who drink at levels that present the greatest risk. Recent clinical studies and demonstration projects show that interventions by obstetric caregivers can help reduce drinking even among women who consume alcohol at the heaviest levels. Brief interventions and motivational interviewing are two approaches that can be adapted for busy medical offices to provide interventions before, during, and after pregnancies. By combining these interventions with a stepped-care approach, practitioners will be able to intervene to prevent drinking during pregnancy while minimizing costs to the patient and demands for limited clinic resources.

The risks associated with drinking during pregnancy are well documented, as evidenced by the other articles in this issue of *Alcohol Research & Health*. Television and radio public service messages, warning labels, and educational campaigns aimed at informing the public about the harm caused by fetal alcohol exposure have led many women to quit or reduce their drinking before or during pregnancy (Waterson and Murray-Lyon 1990). These universal prevention efforts, however, have been largely ineffective in reaching women who drink at levels that present the greatest risks for damaging the fetus (Hankin 1994; Stratton et al. 1996). Community-wide and multi-level strategies for reaching women who drink at the heaviest levels are needed to reduce the incidence of Fetal Alcohol Syndrome and other alcohol-related neurodevelopmental disorders (Smith and Coles 1991). One approach that shows promise for reaching women at risk is the integration of alcohol counseling into gynecologic and obstetric care. Intervening as part of gynecologic and obstetric care enables health care practitioners to reach women before they conceive, during pregnancy, and as part of postpartum care. Such intervention is especially important for pregnant women who would not consider alcohol treatment, but by virtue of their drinking habits (e.g., weekend “binges”) would be placing their unborn babies at risk for alcohol-related impairment.

Several clinical studies and demonstration projects have shown that women can be successfully engaged in efforts to decrease their drinking when approached during routine obstetric care. Moreover, follow-up evaluations of babies born to mothers who reduced their drinking during their pregnancies have revealed better infant outcomes. Based on our review of the effectiveness of interventions held in prenatal clinics, this article presents information on adapting brief motivational approaches for alcohol interventions during prenatal health care and provides a specific model for intervening.

## Methodology

To review the effectiveness of alcohol interventions held in prenatal clinics, we selected 22 studies according to the following criteria. The intervention had to be conducted in a prenatal care setting or in conjunction with a prenatal care intervention. The study had to include a clear measurement of drinking. In addition, the study had to consider a variety of outcome variables to determine the effectiveness of the treatment in reducing alcohol-related harm: alcohol use, treatment retention, pregnancy outcome, and infant outcome. Randomized controlled trials, demonstration projects with some comparison data, and demonstration projects without comparison data were included. We excluded studies that measured abstinence from all substance use as the only outcome variable or that dealt with “alcoholism,” but did not measure alcohol use. Demonstration projects without comparison data which did not demonstrate that a significant proportion of participants were drinking also were excluded, because the conclusions about the effect of the treatment on drinking were too tenuous to be interpreted. (For specific information on the studies examined here, see sidebar.)

**Methods Used in Selecting Studies**

The studies reviewed here include a wide range of treatment approaches, screening and recruitment criteria, gestational periods, settings, outcome variables, and followup periods (see table). Researchers recruited most of the study participants when they were receiving prenatal care from either hospital-based programs or maternal health clinics. Participants were generally selected based on alcohol use. Investigators also selected women who drank at moderate to excessive levels as well as women who currently were experiencing alcohol-related problems. In some instances, treatments were compared in general obstetric populations or in women at risk for reasons other than drinking (e.g., women who smoked or were unmarried). Most of the recruited women were not enrolled in formal alcoholism treatment, although four studies reported on women who were participating in a combined obstetric and substance abuse treatment program.

Thirteen of the studies provided a description of a single treatment intervention and included data describing the outcome of women who participated in the project. Studies of that design are described here as demonstration projects. Two of these reported comparative data of women who refused treatment with women who attended treatment (Eisen et al. 2000; Whiteside-Mansell et al. 1999). Another study of women who were enrolled in a single treatment program compared those who had comorbid conditions with those who did not (Brems and Namyniuk 1999). Interventions in the demonstration projects typically were described in general terms (e.g., counseling, education, substance abuse treatment, or disease model education) or by referring to a specific treatment format (e.g., day treatment, residential treatment, or home visits).

***Demonstration Projects***

Demonstration projects have made major contributions to the study of drinking during pregnancy and its prevention. These projects have shown that women in prenatal care settings can be screened and recruited for treatment by their health care providers and that women often reduce their drinking during pregnancy (e.g., Little et al. 1985; Meberg et al. 1986; Higgins et al. 1995). The demonstration projects also show that offspring of women who reduce their drinking have a lower incidence of fetal alcohol effects than women who continue drinking throughout their pregnancy (Little et al. 1984).

In gathering this body of research, the investigators were creative in the methods they used to recruit women, were flexible in the times during gestation that women were treated, and were thorough in the diverse ways in which they measured outcomes. Additionally, demonstration projects measured the variability of women who participated in treatment and their drinking-related outcomes (i.e., younger women may cut back their alcohol use more than older women) (Rosett et al. 1978). Because of the design limitations (i.e., the lack of control groups), however, these demonstration projects do not allow clear conclusions regarding the efficacy of the treatments used.

***Controlled Trials***

The main weaknesses of the literature on alcohol treatment within prenatal services are the lack of control groups in the majority of reports and the small number of well-controlled trials. Significant proportions of women in the demonstration projects decreased their drinking, but the outcomes cannot be attributed directly to the treatments. Because many women decrease or quit drinking on their own during pregnancy (Kaskutas and Graves 1994), the absence of comparison groups in most of these studies makes it difficult to discern the efficacy of the treatments. In addition, the high dropout rates and the low numbers of women drinking at the heaviest levels in some of these studies render the findings unconvincing as prevention strategies.

Nine of the studies used methods to equate the groups (i.e., random assignment and cohort design), thereby allowing stronger inferences to be made regarding the efficacy of the treatments tested. In this group of studies, alcohol interventions ranged from brief education, advice, and self-help manuals to more intensive programs, including general alcohol counseling with case management or supportive counseling. One such investigation found no added benefit to supplementing standard care with a telecommunications network that provided supportive telephone messages, a patient information hotline, peer conference calls, and telephone followups (Alemi et al. 1996). Waterson and Murray-Lyon (1990) found that women who received advice or who received both advice and a video in addition to the written materials did not report drinking any less than those who only received written materials. This finding may indicate that the written materials alone were enough to catalyze change (Waterson and Murray-Lyon 1990).

Other controlled trials found differences between the interventions used. Positive comparisons indicated the benefit of reduced drinking from 10-minute education sessions combined with self-help; one to two home visits; brief interventions; and a motivational intervention, each of which was provided separately in several different samples (Reynolds et al. 1995; Olds et al. 1997; Hankin et al. 2000; Handmaker et al. 1999).

Surprisingly, one investigation on the use of supportive counseling found that more drinking occurred in the intervention group (Meberg et al. 1986). The higher rates of drinking in the intervention group may have been related to pretreatment differences in drinking between groups that were not controlled and the retrospective assessment of the control group who may have under-reported their drinking. In all cases, the interventions demonstrating positive effects on drinking outcome in the prenatal setting occurred outside of a formal treatment program. Furthermore, the interventions were short term, ranging from 10 minutes to two visits.

***Gender and Other Population Differences in Treatment***

The small number of well-controlled trials reporting on the treatment of alcohol problems in women and pregnant women requires us to interpret the findings of this review with caution. Many treatments have been tested both in male and primarily male samples, but important epidemiological issues distinguish female problem drinkers from male problem drinkers. Differences also exist between pregnant drinkers and women who seek treatment when they are not pregnant.

Distinctions in alcohol use between men and women include what qualifies as safe drinking levels, the prevalence of alcohol problems, and the pattern of heritability (National Institute on Alcohol Abuse and Alcoholism 2000). Differences also can be found among women who seek alcohol treatment. In general, pregnant drinkers who seek treatment tend to be younger and experience fewer alcohol-related problems than women who seek treatment when they are not pregnant (McClelland 1985). The differences between these two populations may necessitate different treatments for women who are pregnant versus those who are not. However, the results of the studies reviewed here appear consistent with the broader treatment literature, which shows that brief interventions and motivational interventions have strong track records for reducing alcohol consumption by both problem drinkers and dependent drinkers (Miller et al. 1998).

Some aspects to treating women who are pregnant are unique to this population, however. Dvorchak and colleagues (1995) cited transportation problems, limited financial resources, and lack of available child care as barriers to treatment among pregnant women. Additionally, Simons and colleagues (2000) stress the importance of addressing the issue of domestic violence and related trauma when counseling women about substance abuse. Although these observations may seem intuitive, no comparisons using an experimental design have been made between treatments addressing these issues and treatments focusing only on drinking. The disparity between speculative theories about treating pregnant women and actual findings clearly indicates that more work is needed to test the ideas that have been proposed in the literature.

—Nancy Sheehy Handmaker and Paula Wilbourne

ReferencesAlemiFStephensRCJavalghiRGA randomized trial of a telecommunications network for pregnant women who use cocaineMedical Care3410SupplOS10OS201996884393310.1097/00005650-199610003-00002BremsCNamyniukLLComorbidity and related factors among ethnically diverse substance using pregnant womenJournal of Addictions & Offender Counseling19276871999ChangGGoetzMAWilkins-HaugLBermanSA brief intervention for prenatal alcohol use: An in-depth lookJournal of Substance Abuse Treatment18436536920001081231010.1016/s0740-5472(99)00105-1CorseSJSmithMReducing substance abuse during pregnancy: Discriminating among levels of response in a prenatal settingJournal of Substance Abuse Treatment1554574671998975100510.1016/s0740-5472(98)00027-0DvorchakPAGramsGTateLJasonLAPregnant and postpartum women in recovery: Barriers to treatment and the role of Oxford House in the continuation of careAlcoholism Treatment Quarterly133971071995EisenMKeyser-SmithJDampeerJSambranoSEvaluation of substance use outcomes in demonstration projects for pregnant and postpartum women and their infants: Findings from a quasi-experimentAddictive Behaviors25112312920001070832710.1016/s0306-4603(98)00116-6ErsohoffDHAaronsonNKDanaherBGWassermanFWBehavioral, health and cost outcomes of an HMO-base prenatal health education programPublic Health Reports98653654719836419268PMC1424500GrantTMErnstCCStreissguthAPPhippsPGendlerBWhen case management isn’t enough: A model of para-professional advocacy for drug- and alcohol-abusing mothersJournal of Case Management531119968715695HalmesmakiEAlcohol counseling of 85 pregnant problem drinkers: Effect on drinking and fetal outcomeBritish Journal of Obstetrics and Gynaecology95324324719883370196HandmakerNSMillerWRManickeMFinding of a pilot study of motivational interviewing with pregnant drinkersJournal of Studies on Alcohol60228528719991009196810.15288/jsa.1999.60.285HankinJSokolRCanestrelliJShernorrNProtecting the next pregnancy: I. Impact on drinking during the subsequent pregnancyAlcoholism: Clinical and Experimental Research24Suppl103A2000HigginsPGCloughDHWallerstedtCDrug-taking behaviours of pregnant substance abusers in treatmentJournal of Advanced Nursing2234254321995749960810.1046/j.1365-2648.1995.22030425.xKaskutasLAGravesKRelationship between cumulative exposure to health messages and awareness and behavior related drinking during pregnancyThe American Journal of Health Promotion9211512419941015071210.4278/0890-1171-9.2.115LarssonGPrevention of fetal alcohol effectsActa Obstetrica et Gynecologica Scandinavica62171178198310.3109/000163483091557866868968LittleREYoungAStreissguthAPUhlCNPreventing fetal alcohol effects: Effectiveness of a demonstration projectCiba Foundation Symposium1052542741984656398910.1002/9780470720868.ch15LittleREStreissguthAPGuzinskiGMAn evaluation of the pregnancy and health programAlcohol Health & Research World1014471198510275265MasisKMayPAA comprehensive local program for the prevention of fetal alcohol syndromePublic Health Reports106548448919911910181PMC1580308McClellandATGuide to the ASI: Background, Administration and Field Testing ResultsRockville, MDNational Institute of Drug Abuse1985MebergAHalvorsenBHolterBModerate alcohol consumption—need for intervention programs in pregnancy?Acta Obstetrica et Gynecologica Scandinavica658861864198610.3109/000163486091570393825527MillerWRAndrewsNWilbournePLBennettMAWealth of alternatives: Effective treatments for alcohol problemsMillerWRHeatherNTreating Addictive BehaviorsNew YorkPlenum Press1998203216National Institute on Alcohol Abuse and Alcoholism10th Special Report to the U.S. Congress on Alcohol and Health: Highlights from Current ResearchU.S. Department of Health and Human Services2000OldsDLEckenrodeJHendersonCRJrLong term effects of home visitation on maternal life course and child abuse and neglect: Fifteen year follow-up of a randomized trial [comment]Journal of the American Medical Association278863764319979272895ReynoldsKDCoombsDWLoweJBPetersonPLGayosoEEvaluation of a self-help program to reduce alcohol consumption among pregnant womenThe International Journal of Addictions304427443199510.3109/108260895090487357607777RosettHLQuelletteEMWienerLOwensETherapy of heavy drinking during pregnancyObstetrics and Gynecology51141461978619335RosettHWeinerLEdelinKCTreatment experience with pregnant problem drinkersJournal of the American Medical Association249152029203319836834593SimonsLDucetteJStahlerGJKirbyKShipleyTEAn Evaluation of a Model of Biopsychosocial Factors That Mediate the Relationship Between Childhood Abuse and Substance Use: Treatment Implications for WomenPaper presented at the College of Problems of Drug Dependence annual meeting in San JuanPuerto RicoJune 2000WatersonEJMurray-LyonIMPreventing fetal alcohol effects: A trial of three methods of giving information in the antenatal clinicHealth Education Research: Theory & Practice553611990Whiteside-MansellLCroneCCConnersNAThe development and evaluation of an alcohol and drug prevention and treatment program for women and children: The AR-CARES programJournal of Substance Abuse Treatment16326527519991019474410.1016/s0740-5472(98)00049-x

## Intervening During Obstetric Care

Despite the evidence that women will engage in alcohol counseling when it is offered as part of their prenatal care, few obstetric practitioners routinely screen, assess, and counsel patients about problem drinking (Morse and Hutchins 2000). The reasons obstetricians frequently cite for not intervening include their lack of time, training, and resources, as well as resistance by the patients themselves. However, as discussed below, brief interventions and motivational interviewing are two methods that address health care practitioners’ concerns and show promise for overcoming these obstacles to intervening.

### Brief Interventions

Routine screening is an essential step toward identifying drinking among pregnant women (Morse and Hutchins 2000). Once a woman is identified as a drinker, health care practitioners are faced with the challenge of how to intervene appropriately. Brief alcohol counseling—that is, one to three patient consultations held in primary health care settings with personalized feedback on health problems and risks, advice, and options for treatment and self-help—have consistently shown significant reductions in problem drinking when compared to no counseling (Bien et al. 1993; Miller 2000). Other benefits of brief alcohol interventions as part of health care have been improvements in alcohol-related health problems (e.g. liver disease), decreased morbidity, and increased adherence to alcohol treatment (Bien et al. 1993). Somewhat surprisingly, brief interventions consistently show outcomes for problem drinking similar to more extended treatment and these changes can be relatively enduring, lasting up to a year or longer (Bien et al. 1993; Miller 2000).

Recent studies of brief interventions have demonstrated their feasibility for reducing alcohol consumption among pregnant drinkers. Hankin and colleagues (2000*a*) conducted a randomized controlled trial to examine the effect of two brief intervention strategies on drinking in subsequent pregnancies. Women who reported drinking during pregnancy were randomly assigned to receive either the brief intensive intervention or a control condition of a standard warning about antenatal drinking. The control group intervention was described as using encouraging statements such as, “You can have a healthier baby if you cut back or stop drinking during pregnancy.” Participants then were followed into their subsequent pregnancies. The group that received the intensive intervention was offered brief “booster” sessions during the subsequent pregnancy. Although the intensive brief intervention group was drinking about the same amount in the second pregnancy as the first pregnancy, women in the control group were drinking almost twice as much as they consumed during the first pregnancy. Thus, the benefits of the brief, but intensive intervention apparently dampened the rise in potential fetal alcohol exposure levels during subsequent pregnancies. Furthermore, the study found that women who reported the heaviest prepregnancy drinking showed the largest reduction in drinking following the brief intensive intervention. More importantly, the study found that babies born to women in the brief intensive intervention groups showed better growth outcomes at birth (Hankin 2000*b*).

Chang and colleagues (2000) investigated whether adding a brief intervention to standard care would increase abstinence rates among a sample of pregnant outpatients. The intervention focused on setting drinking limits and problem-solving about how to avoid drinking in risky situations. Most patients who set abstinence as their drinking goal at the beginning of their prenatal care either remained abstinent or significantly reduced their alcohol consumption. This outcome was positively correlated to the patients’ concerns about the effect of drinking on their babies. Women who reported that their reason for change was apprehension about the effects of fetal alcohol exposure drank significantly less at followup than the other participants.

### Motivational Interviewing

In the absence of extensive alcohol treatment, an explanation for the success of brief interventions is that they increase the patient’s readiness for change. Motivational interviewing is an empathic patient-centered counseling approach for increasing readiness by resolving ambivalence about behavior change (Miller and Rollnick 1991). The process involves the exploration of the patient’s ambivalence (i.e., the “pros” and “cons” for drinking) in an atmosphere of acceptance, warmth, and regard. Although the session is directive, direct persuasion and coercion are avoided. A goal is to enhance the discrepancy between the reasons for changing (e.g., risks of brain damage to the fetus) versus staying the same (e.g., not giving up drinking friends). Important qualities of an effective interviewer are maintaining an optimistic attitude about change, having a compassionate style, and avoiding arguments or evoking patient defensiveness (Miller and Rollnick 1991).

More than 24 studies of motivational interviewing have yielded beneficial effects in decreasing problem drinking, drug addiction, marijuana abuse, diabetes management, smoking, and cardiovascular rehabilitation (Miller 2000). Many studies have used motivational interviewing as a stand-alone intervention rather than as an addition to more extensive clinical treatment. The specific format of motivational interviewing has varied in length from a single counseling session, and a two-session assessment and feedback approach, to the four-session Motivation Enhancement Therapy (Project MATCH 1997). Clinical studies show that motivational interviewing has been as effective in reducing drinking and related problems as more extensive alcohol treatments such as Cognitive-Behavioral Therapy and 12-Step Facilitation, and consistently yields beneficial and relatively lasting effects (Project MATCH 1997).

Health care practitioners are likely to see women who are ambivalent about abstinence. Those women often either are unaware that their level of alcohol consumption presents a risk to the fetus, or they recognize that drinking is a problem but have not committed to abstinence. Offering premature advice or making referrals to alcohol treatment is likely to be ineffective, creating instead a defensiveness among women who are undecided about whether the costs of drinking outweigh the perceived benefits, or who are uncertain about whether they can change (Miller and Rollnick 1991). Researchers have found that when interviewers exert more pressure or present intellectual arguments, clients tend to react more defensively. The degree of defensiveness or resistance that a patient exhibits during a session has been shown to be a predictor of poorer drinking outcomes, and researchers have found that an empathic therapist style was predictive of decreased patient resistance (Miller et al. 1993).

Several National Institute on Alcohol Abuse and Alcoholism-funded research programs are underway to evaluate the benefits of motivational interviewing with pregnant problem drinkers. One study has reported findings on a pilot study of these methods for pregnant drinkers (Handmaker et al. 1999*b*). Following completion of a screening questionnaire, pregnant women who reported any recent alcohol consumption were randomly assigned to either a motivational interview or an information-based intervention. The information-based intervention was a personalized letter cautioning that drinking was known to be hazardous and recommending that the participants talk about this with their obstetric care practitioners. The goal of the motivational interviewing session was to facilitate a decision to change by gently guiding the participants to weigh their drinking against the risks. A key strategy toward facilitating a decision to abstain was exploring and resolving the participants’ ambivalence about decreasing their drinking. The health of the unborn baby was a major motivational theme, although direct assessment of the impact of drinking on the baby’s health was not available. Instead, a gestational chart illustrating fetal development at critical periods was incorporated into the motivational interviewing session. The interview proceeded with open-ended questions (e.g., “What do you know about the effects of drinking during pregnancy?”) to evoke concerns related to the risks associated with fetal alcohol exposure and empathic reflections of the participant’s responses (e.g., “You want your baby to have the best chance at life”) to reinforce talk about change. As in Chang’s study, counselors helped the women explore alternatives to drinking, especially for high-risk situations (e.g., not drinking at a party) and helped them generate their own ideas about maintaining abstinence, including engaging in alcohol treatment. Results showed both the treatment (i.e., motivational interview) and control (i.e., caution plus referrals) groups significantly decreased their alcohol consumption at the followup. The study found a differential response, however, to the motivational interview in women drinking to high doses, as estimated by peak blood alcohol concentration (BAC)^1^. Women who had been reaching high BACs before the motivational interview were drinking at significantly lower levels at followup compared to women in the control group. That is, the women in the treatment group either were extending their alcohol consumption over longer periods or they consumed less alcohol during a drinking episode. Thus, women who were placing their unborn babies at the greatest risk, based on estimated doses of alcohol exposure, responded favorably to the motivational intervention. These findings are preliminary. Moreover, the use of average metabolism rates to calculate measures of BACs is not exact because of individual differences in metabolism rates. However, the outcomes found among the heaviest drinkers are consistent with the literature on motivational interventions (Hankin et al. 2000*a*; Miller 2000).

An interesting finding from the pilot study of motivational interviewing seen in other studies of brief interventions is that the assessment process itself may lead to a reduction in drinking. It is plausible that assessment methods conducted in a reflective, nonjudgmental interviewing style may increase awareness and problem recognition, processes known to promote behavior change. This potential effect of screening and assessment among female participants has been replicated in other studies (e.g., Scott and Anderson 1990).

### Comprehensive Care

Reviews of treatment programs for pregnant women who use alcohol or drugs suggest that comprehensive care which coordinates medical with alcohol and drug treatment and social services is most effective (Finkelstein 1993). This is particularly true for women who drink at the heaviest levels, who are likely to be smoking or using illicit drugs, to be socioeconomically disadvantaged, or to have comorbid depression or other psychological distress. Comprehensive care programs vary in treatment modalities and services, but components such as group or individual therapy, detoxification, case management, parenting classes, and self-help frequently are included. In the absence of clinical trials comparing comprehensive care with the alternative, less-intensive approaches, such as brief interventions and motivational interviewing, researchers cannot determine which patients need comprehensive care and which components of care are essential. In the next section, we propose a stepped approach to intervening should a patient need more than a motivational interview or brief intervention.

## A Stepped Care Model for Prenatal Settings

A “one-stop shopping” concept in which social workers, psychiatrists, case managers, and psychotherapists work collaboratively as part of a multidisciplinary team within obstetric care is the ideal when caring for the addicted pregnant patient (Tanney and Lowenstein 1997; Finkelstein 1993). However, most prenatal programs (e.g., private practices, rural health care, and stand-alone out-patient obstetric clinics) are not prepared to offer such comprehensive and integrated care. A feasible alternative is the provision of brief interventions, referrals for other services, and monitoring, which can lead to reductions in drinking among pregnant women as well as to increases in adherence of referrals to alcohol and drug treatment and other support services.

A recent approach to decisionmaking about alcohol treatment known as “stepped care” applies decision rules derived from other areas of health care to the alcohol treatment field (Sobell and Sobell 2000). According to this approach, alcohol treatment that is individualized, consistent with state-of-the-art literature, and the least restrictive, is likely to work. This approach emphasizes “serving the needs of clients efficiently, but without sacrificing the quality of care” (Sobell and Sobell 2000, p. 578). Stepped care is consistent with health care delivery for other health problems and minimizes costs and demands for limited resources. Used within a network of comprehensive services, stepped care also reduces the demands on female patients for child care, transportation, and expenses for healthcare, which women frequently mention as obstacles to treatment.

Stepped care begins with broad, sensitive screening that includes brief self-administered questionnaires like the five-item TWEAK, which has demonstrated sensitivity and specificity for problem drinking among pregnant women (Stratton et al. 1996). A model for intervening with the pregnant substance-using woman is illustrated in the figure below. This model proposes the use of broad, sensitive screening in prenatal clinics and, for those who report either drinking during pregnancy or alcohol-related problems in the past year, a more thorough assessment interview conducted in an empathic style. The next step may be a second assessment, combined with advice. This step may suffice for lighter drinkers and also would identify the heavier, high-risk drinkers who need brief intervention and monitoring. The third step is a motivational intervention with a health care professional, during which the patient and counselor might negotiate a plan for change. Plans for change can be any combination of options that will support sobriety, such as specialized alcohol treatment, self-help, community resources, case management, and financial assistance.

Heavy drinking also is likely to be accompanied by comorbid conditions of depression, anxiety, and other psychological problems as well as concomitant drug use, particularly cigarette smoking. High rates of posttraumatic stress disorder and histories of sexual abuse frequently are reported in female substance-abusing populations. As a result, matching patients with treatment to meet specific needs, such as mental health care with a substance use component, is recommended. Family histories of drinking among female relatives and drinking among significant others have been correlated with problematic drinking (e.g., Handmaker et al. 1999*b*; Stratton et al. 1996). Consequently, strategies that include family members are likely to improve outcomes. Ideally the prenatal care setting would develop a network with other services for referral as well as monitor progress and make new referrals if previous actions were not helpful in reducing harm.

## Future Directions

Most medical schools and continuing medical education courses offer minimal training, if any, in alcohol counseling. Health care practitioners need practical strategies for brief patient consultations that will foster compliance with abstinence and encourage participation in alcohol treatment when necessary. A feasibility study of the use of videotaped instruction as a method for improving the efficacy of brief counseling among health care practitioners demonstrated one possible strategy (Handmaker et al. 1999*a*). In that study, health care practitioners were randomly assigned to view either a videotaped training based on motivational interviewing or a docudrama about the effects of fetal alcohol syndrome. Results showed that the practitioners who viewed the docu drama demonstrated a more confront ational style in role-played sessions following the video than those who viewed the skills-training videotape. Although the health care practitioners who viewed the counseling training tape were not proficient in motivational interviewing skills after one session, they appeared to direct the consultation more effectively toward a decision to change. These health care practitioners demonstrated a nar row set of skills shown in the videotape that included developing a discrepancy between reasons for change and not changing, being empathic, supporting the belief in the patient’s ability to change, and minimizing confrontation. Ongoing booster sessions or guided experiences in addition to videotaped training might lead to increased proficiency.

## Conclusions

Most studies of integrated alcohol treatment with prenatal care have been limited by the lack of control groups, small numbers of heavy drinkers, and inability to separate the effects of treatment from naturally occurring change during pregnancy. Another limitation is the general lack of confidence in the outcome measures, which rely primarily on self-report.

Demonstration projects have shown that women can be screened for their drinking by their providers in prenatal care settings. Controlled trials found that even brief interventions produce positive results. Brief interventions and motivational interviewing are two ways obstetric care providers can intervene with pregnant women who continue to drink. Both these methods may be applied through a stepped care approach that can serve the needs of clients efficiently without sacrificing quality of care. By applying decision rules derived from other areas of health care, practitioners can minimize costs and demands for limited resources.

Researchers have recommended embedding alcohol and drug use within the context of broader efforts toward health and well-being. Continuing to educate the public about how to inter vene with family members and using media campaigns to encourage women to discuss alcohol use in health care set tings may be particularly advantageous.

Family counseling, which has been shown empirically to increase engagement and retention of resistant problem drinkers and drug users (Smith et al. 1999), is a yet untested direction for treatment of pregnant populations. Further study is also necessary to learn the best treatment for female problem drinkers and to discern any differences between pregnant women and those who seek treatment when they are not pregnant. In addition, further study of methods to increase the effectiveness of health care practitioners in brief interventions and motivational interviewing is needed.

## Figures and Tables

**Figure:**
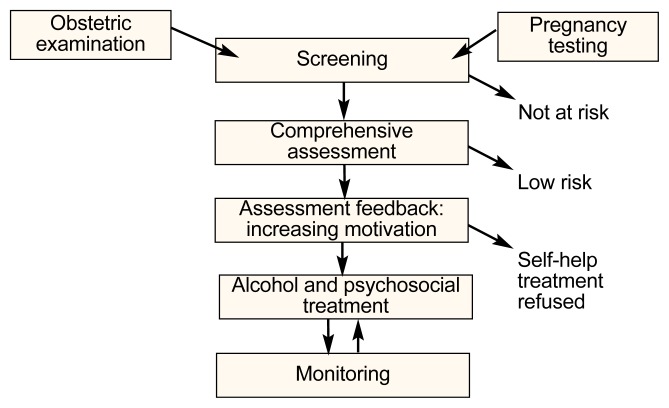
A stepped-care model for intervening with pregnant women who are using alcohol or other drugs.

**Table:** Studies of Interventions for Preventing Alcohol-Related Birth Defects

Source	Setting	Criteria	Screened	Identified or Reported	Intervention	First Assessment	Followup Assessment (*n*)	Results
Rosett et al. 1978	Hospital-based PNC	Moderate and heavy drinking	322 participants	42 heavy drinkers	General alcoholism counseling and PNC	Start of PNC	Birth (42)	Older women and those with a greater number of pregnancies attended less PNC. Heavier drinking women had smaller infants with more anomalies.
Ersohoff et al. 1983*+	HMO-based PNC	Smoking during pregnancy	236 women screened	129 smokers—Cohort 1: 72; Cohort 2: 57	Cohort 1: standard PNC; Cohort 2: health counseling, smoking cessation, and standard PNC	Before 24 weeks’ gestation	2 months’ postpartum (129)	Extremely low rates of drinking were reported, with no difference between groups. A trend for reduced smoking related to the intervention was reported. Infants born to women in the intervention group had higher birth weights.
Rosett et al. 1983	Hospital-based PNC	Drinking 45 drinks per month, with 5 or more drinks on some occasions	162 heavy drinkers	49 attended 3 or more visits	Counseling during regular PNC visits, abstinence goal, referral to AA, counseling for other health problems	Start of PNC	Unclear (49)	Young women with their first pregnancies showed the largest reductions in drinking. Women who primarily used alcohol reduced their drinking less than those who smoked and used drugs.
Larsson 1983	Maternal health clinics	Drinking greater than 30 grams per day during past month	464 screened	50 heavy or excessive drinkers	NA	Start of PNC	Birth (464)	No differences were found in OB complications across drinking levels.
Little et al. 1984	Referral from screening in PNC clinics and phone hotline	Excessive drinking or alcohol-related problems	1,126 pregnant women making contact with program	304 seen in program	AA, general alcoholism counseling, home visits, case management, PNC, developmental assessments	During pregnancy	6 months’ postpartum: 151 infants; 304 women	Women who reduced their drinking had fewer cases of FAE. The longer that women were in treatment the less they drank.
Little et al. 1985	Referral from screening in PNC clinics and phone hotline	Moderate alcohol problems	1,265 screened	107 moderate drinkers	AA, general alcoholism counseling, home visits, case management, PNC, developmental assessments	During pregnancy	Birth (107)	Women reduced their drinking throughout their pregnancy. The heaviest drinkers had the smallest babies.
Halmesmaki et al. 1988	Hospital-based PNC	Problem drinking	85 pregnant problem drinkers	85 pregnant problem drinkers	General alcohol counseling	Start of PNC	Birth (85); 6 mo (72); 12 mo (47)	Most women reduced their drinking. FAE was seen in 42 infants, and FAS was seen in 20 infants.
Waterson and Murray-Lyon 1990	PNC provider	All pregnant women; about 36% were drinking one drink per day or more	2,100	756 women drinking one or more drinks per day before pregnancy	Group 1: written information Group 2: information plus advice Group 3: information, advice and a video	Start of PNC	28 weeks’ gestation (1,145); birth (1,134)	No difference in the number of women drinking above the “recommended” safe limit of seven drinks per week in any intervention group. Advice and video were not shown to be better than written material alone.
Masis and May 1991	Indian Medical Center	Any drinking	48 referrals	39 contacts	General alcoholism counseling, case management, and counseling regarding contraception	During pregnancy	18 months’ postpartum (32)	Most women chose a form of reliable birth control; 46% were abstinent at followup.
Higgins et al. 1995	Integrated PNC and substance abuse treatment program	Enrollment in substance abuse treatment; 71% drinking alcohol	60 available in program	34 consented	PNC and substance abuse treatment	Start of PNC	Birth (31)	Six women decreased their alcohol use, 13 stopped drinking completely, and 0 did not change their drinking behavior.
Reynolds et al. 1995*+	Public health maternity clinics	Drinking in the past month	1,201 screened	78	Group 1: standard treatment; Group 2: standard plus 10-minute education session and self-help manual	Start of PNC	After birth (72)	Trend found (*p*<0.058) for higher “quit rate” in the intervention group.
Alemi et al. 1996*+	Women’s health clinic	Drinking three times per week and using cocaine	179	179	Group 1: standard treatment; Group 2: standard plus a telecommunications intervention	Third trimester of pregnancy	6 months’ postpartum (160)	No statistical difference found between treatment and control groups on alcohol use.
Grant et al. 1996	Hospital and community service referral	Heavy drug/alcohol use	151	151	One-to-one management	38 weeks’ gestation	12 months’ postpartum (51)	41 started substance abuse treatment; 80% were drinking at delivery, and 71% were drinking 12 months later.
Meberg et al. 1986*	Referral from medical provider	Light to moderate drinkers recruited for intervention	not reported	132, 74 light to moderate drinkers and 58 consecutive deliveries used as control subjects	Group 1: Supportive counseling; Group 2: Consecutive admissions recruited at delivery	Late first or early second trimester, near the start of PNC	Delivery	All women in the study reduced their drinking. More women in the intervention group reported the use of alcoholic beverages. This finding may be due to differences in assessment between the two groups
Olds et al. 1997*+	PNC setting	Women at risk: women with their first pregnancies who were < age 19, unmarried, or from low socio-economic status	500 asked to participate	400 consented	Group 1: standard treatment; Group 2: standard plus prenatal home visit; Group 3: standard plus one prenatal and postpartum home visit	Before third trimester of pregnancy	Age 15 (324)	Two intervention groups did not differ from each other. Women who received home visits reported fewer alcohol- and drug-related problems than those who received only standard treatment.
Corse and Smith 1998	Integrated PNC and substance abuse treatment program	Heavy drinking	77 enrolled participants	77 enrolled participants	Group and one-to-one counseling	During pregnancy	6 months’ postpartum (77)	50.6% largely abstinent; 35.1% somewhat reduced; 14.3% no change.
Whiteside-Mansell et al. 1999*	Substance abuse treatment program with integrated PNC	Pregnant and parenting women in substance abuse treatment	95 eligible	72 participants; 23 refused treatment	Disease model and education-based day treatment with PNC and health education	Third trimester	Birth (27 participants and 10 non-participants), 6, 12, and 18 months	Treatment participants made “larger reductions” in drinking, had less preterm labor, and had fewer infections. No differences in developmental outcomes between groups.
Brems and Namyniuk 1999	Residental drug treatment program	Enrolled in residental treatment	192	Compared comorbid women with non-comorbid women within the sample	Residental treatment	During treatment	End of treatment (192)	Treatment retention: comorbid women were 2.65 times more likely to leave within 14 days of admission than non-comorbid women; higher MAST scores in comorbid than noncomorbid women (5.25 vs. 4.65).
Hankin et al. 2000*+	Hospital after delivery of alcohol-exposed infant	Risky drinkers who delivered an alcohol- exposed infant	96 recruited	96 recruited	Group 1: brief intervention (*n*=72); Group 2: physician’s advice (*n*=24)	Birth of previous child	13 months’ postpartum; birth of second infant	Women receiving the brief intervention drank less during their second pregnancy.
Eisen et al. 2000*	Nine maternal health clinics	Pregnant women reporting alcohol or drug use	658	658	Group 1: case management and referral or day treatment; Group 2: those declining services	Start of PNC	30 days’ postpartum (398); 6 months’ postpartum (257)	Women who drank at the first assessment were more likely to drop out by the 6-month assessment. More participants reduced their drinking at both followups than those declining services.
Chang et al. 2000*+	Hospital-based PNC setting	T-ACE positive	250 T-ACE positive women recruited into study	123 treatment; 127 control	Group 1: standard PNC; Group 2: standard plus brief intervention and pamphlet	Start of PNC (about 16 weeks)	Postpartum (248)	Women receiving brief intervention were more likely to remain abstinent after stopping drinking early in their pregnancy.

AA = Alcoholics Anonymous; HMO = health maintenance organization; NA = not applicable; *p* = significance; PNC = prenatal care.

* Some comparison data are available.

+ Some methods to equate groups are employed, either cohort design or random assignment.

## Footnotes

1 For each drinking day, the estimated number of drinks, the alcohol content of the drinks, and the length of drinking episodes were obtained. All alcohol consumption was converted into ethanol units equal to 0.5 oz. (15 mL) of absolute alcohol. Using the aforementioned data, the recent weight of the participants, and an average rate of alcohol metabolism for women, computer projections of BAC peaks were calculated.
